# Acceptance, availability, and feasibility of RTS, S/AS01 malaria vaccine: A review

**DOI:** 10.1002/iid3.899

**Published:** 2023-06-14

**Authors:** Hassan Mumtaz, Abdullah Nadeem, Wajeeha Bilal, Farrukh Ansar, Saira Saleem, Qaisar Ali Khan, Tamara Tango, Christopher Farkouh, Naod F. Belay, Ravina Verma, Matthew Farkouh, Muhammad Saqib

**Affiliations:** ^1^ Health Services Academy Islamabad Pakistan; ^2^ Dow University of Health Sciences Karachi Pakistan; ^3^ King Abdullah Teaching Hospital Mansehra Pakistan; ^4^ Mayo Hospital Lahore Pakistan; ^5^ DHQ and Teaching Hospital KDA Kohat Kohat Pakistan; ^6^ Faculty of Medicine Universitas Jakarta Indonesia; ^7^ Rush Medical College Chicago Illinois USA; ^8^ Michigan State University East Lansing Michigan USA; ^9^ St. Georges University Grenada West Indies; ^10^ Ponce Health Sciences University St. Louis Missouri USA; ^11^ Khyber Medical College Peshawar Pakistan

**Keywords:** AS01, malaria, *Plasmodium*, RTS, S, vaccine

## Abstract

**Introduction:**

In malaria‐stricken regions, malaria continues to be one of the primary causes of mortality for children. The number of malaria‐related fatalities has drastically decreased because of artemisinin‐based pharmacological regimens.

**Methods:**

Two independent researchers did a comprehensive literature search using PubMed/MEDLINE and Google Scholar from its inception to September 2022.

**Results:**

After evaluating RTS, S/AS01 for its safety, effectiveness, and feasibility, the European Medicines Agency (EMA) issued a favorable conclusion. It was suggested that the RTS, S malaria vaccine be used extensively by the World Health Organization on October 6, 2021. The successful pilot program testing the malaria vaccine in Ghana, Kenya, and Malawi served as the basis for this proposal.

**Conclusion:**

Several challenges need to be addressed to ensure the success of vaccination programs. From the acceptability perspective, issues such as inadequate community engagement, concerns about side effects, and issues with the delivery and quality of healthcare services can affect the acceptance of the vaccine. From the feasibility standpoint, factors such as lack of transportation or long distances to healthcare facilities and the perception of completion of the vaccination calendar can affect the feasibility of the vaccine. Lastly, the availability of the vaccine is also a major concern as it may not be readily available to meet the demands.

## Graphical abstract

RTS, S/AS01 vaccine offers a manageable route of administration and some evidence of effectiveness against malaria, albeit simultaneously increasing the incidence of meningitis and cerebral malaria complications.

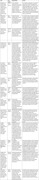

## 1 INTRODUCTION

Infection with a protozoan parasite of the genus of the order Haemosporida in the phylum Apicomplexa causes malaria, the most significant parasitic illness of humans.^1^ The mortality and morbidity rates from *Plasmodium falciparum* malaria continue to be the highest of any malarial species. Up to 87% of people in endemic regions get infected with no outward signs of illness.^2^ Malaria is characterized by a cluster of symptoms including high body temperature, shivering, headache, diarrhea, nausea, vomiting, pain in the muscles and joints, and rapid breathing and heart rate. With varying degrees of success, attempts have been conducted over the past century to control, decrease, and eventually eradicate the effects of malaria, particularly in tropical and subtropical Africa and some regions of Asia.^1^ Over the last two decades, researchers have made significant strides toward creating a malaria vaccine.

As of 2021, the global incidence of malaria has been estimated to have reached a total of 247 million cases across 84 nations where the disease remains endemic. This represents an increase of 2 million cases when compared to the previous year. It is imperative to note that the continued presence of malaria in these regions highlights the ongoing need for effective prevention and treatment measures to combat the spread of this debilitating illness.^3^ The African region accounts for about 95% of cases and 96% of deaths globally due to malaria. Approximately 80% of all deaths due to malaria in Africa occurred among children under the age of 5 years in 2021. Although this mortality is alarming, it has relatively decreased from the 91% it was back in 2000.^4^ The situation is further exacerbated by the decline in funding for malaria control and elimination efforts. Efforts must be redoubled to address these challenges, to mitigate the impact of malaria on vulnerable communities.^5^ The RTS, S vaccine, also known as Mosquirix, is a pioneering development in the fight against malaria. It is the first and currently the only vaccine that has been demonstrated to significantly reduce the occurrence of malaria in children, including severe cases, hospital admissions, and the need for blood transfusions. The vaccine is currently being implemented in areas where malaria transmission is moderate to high and is responsible for a significant proportion of childhood visits to healthcare facilities, often accounting for up to 60%.^6^ The launch of the pilot program for the vaccine in Ghana, Kenya, and Malawi has been met with strong community demand and has been able to reach a significant number of children in a relatively short period of time. To date, more than 1.7 million doses of the vaccine have been administered, providing additional malaria protection to over 650,000 children. In a time where global progress in controlling malaria has stagnated, the protection provided by the RTS, S vaccine, when used in conjunction with currently recommended malaria control measures, has the potential to save tens of thousands of lives each year. The widespread adoption and implementation of this vaccine could be a game‐changer in the fight against malaria.^6^

## 2 METHODS

A Boolean search was carried out in MEDLINE‐PubMed, from inception till September 2022, using the search string “(RTS OR RTS, S/AS01) AND (malaria vaccine OR malaria vaccine acceptance OR intention to vaccinate) AND (availability OR malaria vaccine availability)”. Full text of all the related articles in English with supplementary appendices was retrieved. Additionally, the full text of relevant cross‐references was also retrieved. The articles retrieved are enlisted in Table 1. The Inclusion criteria for studies were as follows: (1) Freely accessible, full articles (2) Original studies, observational, cross‐sectional, and randomized controlled trials (3). Only Published studies in peer‐reviewed journals were included for cohorts in stated conflict settings. However, all the reviews, editorials, commentaries, case reports, and case series were excluded. Articles without complete contents or written in languages other than English were not included.

**Table 1:** Summary of the studies on acceptance of RTS, S/AS01 malaria vaccine.

Title	First author	Methodology	Results
Willingness to accept malaria vaccine among caregivers of under‐5 children in Southwest Ethiopia: a community‐based cross‐sectional study^7^	Getachew Asmare	In the months of September 2021, a cross‐sectional survey based in the community was carried out among parents of young children. Interviews with 406 carers of young toddlers were conducted.	The majority of responders, 131 (32.3%), were in favor of immunizing their kids. Marital status (AOR = 1.243; 95% CI 1.021–3.897), knowledge (AOR = 3.120; 95% CI 1.689–5.027), and prior experience with childhood vaccination (AOR = 2.673; 95% CI 1.759–4.101) were all found to be significantly associated with willingness to accept a malaria vaccine for their children, with a *p*‐value of .05.
Stakeholders’ opinions and questions regarding the anticipated malaria vaccine in Tanzania^8^	Sally Mtenga	2123 women with children under five years old completed a structured questionnaire. 12 focus group talks and 46 in‐depth interviews were conducted.	In all, 84.2% of the moms gave the malaria vaccination excellent approval. According to religion, profession, tribe, and location, acceptance differed considerably (*p* = .001). 92% percent of respondents said they will accept the malaria vaccination despite the requirement to continue using insecticide‐treated nets (ITNs), and 88.4% said they will accept the vaccine even if their children contract the disease less frequently than unvaccinated children.
Acceptance of malaria vaccine by a rural community in Nigeria^9^	Maduka D Ughasoro	This cross‐sectional study was conducted in a rural area in south‐eastern Nigeria. A standardized questionnaire was used to interview 156 household heads in total.	The majority (78.2%) agreed that malaria is the most prevalent ailment in the neighborhood, while 55.1% thought the best malaria prevention strategy is presumptive treatment. The majority of research participants (98.7%) inoculated their kids against diseases that can be prevented by vaccines but only 91.6% said they would be open to receiving a malaria vaccine. 35.4% of non‐febrile respondents reported having malaria parasitemia, while 17.9% reported using mosquito nets.
Acceptance of a malaria vaccine by caregivers of sick children in Kenya^10^	David I Ojakaa	During the 2010 Kenya Service Provision Evaluation Study, interviews from a standard questionnaire were done with 2003 carers at 695 randomly chosen health facilities throughout Kenya. STATA was used to conduct a multinomial regression analysis of quantitative data to examine the factors that influence parents’ willingness to vaccinate their children against malaria.	90% of carers who were questioned about bringing their child to the medical facility were mothers, and 77% of carers were between the ages of 20 and 34. Overall, 88% of respondents said they would agree to a malaria vaccination, both for their child and a child in their community. The rate of vaccine approval ranged from 98.9% in the malaria‐endemic Nyanza Province to 23% in the region of North Eastern Province that has seasonal transmission. Only 56% of those who had never attended school said they would accept the vaccine for a child, compared to 94% of respondents who had gone to school at least in part.
Are malaria transmission‐blocking vaccines acceptable to high‐burden communities? Results from a mixed methods study in Bo, Sierra Leone^11^	Kaci D McCoy	This study used a mixed methods approach to understand community knowledge, attitudes, and practices related to malaria and vaccination in general. This included: (i) a population‐based cross‐sectional survey (*n* = 615 adults), (ii) 6 focus group discussions with parents, and (iii) 20 key informant interviews.	According to this study, the majority of adults are open to receiving a TBV vaccination. Most respondents reported having had positive past experiences getting children's and adults’ immunizations against different infectious diseases. The majority of participants in focus groups and interviews thought that community members would embrace vaccination as a component of an integrated malaria control strategy.
Community perceptions of a malaria vaccine in the Kintampo districts of Ghana^12^	Lawrence G Febir	Data collection techniques included both qualitative and quantitative approaches. There were a total of 12 FGDs, 15 IDIs, and 466 household head interviews.	Participants had a general understanding of immunizations. Most respondents wanted all childhood diseases, including malaria, to be vaccinated against for their children.
Awareness, perceptions and intent to comply with the prospective malaria vaccine in parts of South Eastern Nigeria^13^	Uchechukwu M. Chukwuocha	Data were gathered from 500 consenting caretakers who were chosen at random using structured, pretested questionnaires.	The survey indicated that while there is a high level of public knowledge of malaria as a health issue (89.8%), there is a low level of public awareness about a potential malaria vaccine (48.2%). Up to 88.2% of respondents expressed a favorable opinion of the vaccine, of which 65.2% did so strongly. 95.6% of the study group showed high levels of positive intent to cooperate with the impending malaria vaccine.
Factors associated with malaria vaccine uptake in Sunyani Municipality, Ghana^14^	Dennis Tabiri	A total of 424 parents/caregivers and 424 children participated in the study They were drawn from the six sub‐Municipalities in the Sunyani Municipality. The study lasted approximately 10 months	While uptake for the first dose was 94.1%, it reduced to 90.6% for the second dose and to 78.1% for the third dose. RTS,S 1 and RTS,S 2 uptake met the WHO target of 90% however uptake of RTS,S 3 did not.
Factors likely to affect community acceptance of a malaria vaccine in two districts of Ghana: a qualitative study^15^	Arantza Meñaca	The study was conducted in two purposively selected districts: the Ashanti and Upper East Regions. A total of 25 focus group discussions, 107 in‐depth interviews, and 21 semi‐structured observations at Child Welfare Clinics were conducted.	This study has identified several factors that may facilitate the introduction of a malaria vaccine. Malaria was acknowledged to be one of the most common health problems among children. The communities highly valued vaccines and cited vaccination as the main motivation for taking children to Child Welfare Clinics. Nevertheless, knowledge of specific vaccines and what they do was limited.
Complex realities: community engagement for a pediatric randomized controlled malaria vaccine trial in Kilifi, Kenya^16^	Vibian Angwenyi	Detailed one‐on‐one interviews with trial researchers (*n* = 5), community leaders (*n* = 8), parents (*n* = 15 with children enrolled and *n* = 4), and group discussions with fieldworkers (*n* = 6) and facility personnel (*n* = 2) were conducted by social scientists. Around 150 CE activities were observed while surveying the participating families (*n* = 200).	31% (*n* = 62) of those who participated in the activity said it was primarily to study how vaccination may prevent malaria. 2. 15% (*n* = 30) of respondents stated that malaria may still affect their kids. Parents who had participated in the study for a year reported, in 93% of cases (*n* = 186), being satisfied with their choice.
Community perceptions of malaria and vaccines in two districts of Mozambique^17^	Allison Bingham	A qualitative investigation in two districts in southern Mozambique where malaria is prevalent. They performed 26 in‐depth interviews and 23 focus group talks using criterion‐based sampling.	Vaccines are viewed as a way to maintain the health of children and the community at large by lowering the risk of childhood diseases. Long lines, a lack of personnel, and inadequate resources at medical institutions are some of the perceived barriers to receiving immunization services. The adoption of routine childhood vaccinations is positive for the development of a malaria vaccine.
Caregiver and community perceptions and experiences participating in an infant malaria prevention trial of PfSPZ Vaccine administered by direct venous inoculation: a qualitative study in Siaya County, western Kenya^18^	Florence Achieng	Twelve focus groups and 28 in‐depth interviews explored perceptions of the DVI procedure in infants, factors influencing trial acceptability, and barriers to sustained trial participation.	Despite the high levels of support for DVI among parents whose children received the PfSPZ vaccine (or a placebo), early trial withdrawal and unsuccessful completion of trial procedures for some infants were caused by resistance to the trial procedures from other non‐sensitized households and family members.
Community perceptions of malaria and vaccines in the South Coast and Busia regions of Kenya^19^	David I Ojakaa	South Coast and Busia, two malaria‐prone areas of Kenya, were the locations of this qualitative study. There were 20 focus group meetings, 22 in‐depth interviews, and 18 exit interviews done in total.	A malaria vaccine would be well‐received by the community, although there would be some skepticism and concerns. The report brings up concerns that ought to drive communications plans and direct Kenyan policy choices regarding the launch of a malaria vaccine in the future.

## 3 CHALLENGES AND BREAKTHROUGH: A REVIEW OF VACCINE ACCEPTANCE, AVAILABILITY, AND FEASIBILITY

The studies retrieved through the screening process are summarized in Table 1. It enlists the summary of studies based on analyzing vaccine acceptance. The results are thematically elaborated specifically to conflict settings relative to the possible determinants for the findings quoted in Table 1.

### 3.1 Development of malaria vaccine

The WHO's endorsement of RTS, S is a crucial step towards getting the vaccine to children in need, but the limited supplies and slow production rates mean that most children will not benefit soon. It is worth noting that the demand for the vaccine is high, with estimates ranging from 100 million to 160 million doses a year needed just for malaria‐endemic areas in sub‐Saharan Africa, and it will take time for the technology transfer and ramping up of production to meet that demand.^20^ The World Health Organization (WHO) endorsed the first‐ever malaria vaccine in 2021, but efforts to produce and distribute the vaccine have fallen short due to a lack of funding and commercial potential. Initially, the drug maker committed to producing up to 15 million doses annually through 2028, following 2019 pilot programs, but is unlikely to make more than a few million annually before 2026.^21^ The 2015 publication of the trial's results marked an encouraging step forward in creating a malaria vaccine for children in Africa. Clinical and severe malaria cases decreased by one‐third over 4 years in children who got the RTS, S vaccine as part of a three‐dose series followed by a booster dose. Lower vaccine efficacy was seen in young children of age group 6–12 weeks old at the time of first vaccination. Overall, researchers had positive feelings about the safety of vaccines, however, they did note that several red flags, such as febrile convulsions, meningitis, and cerebral malaria, need additional study. This vaccine's ability to protect areas where other effective malaria preventions and treatment interventions were already underway was particularly striking. These interventions included the use of bed nets, antimalarial drugs for disease treatment, residual insecticide spraying in the home to prevent mosquito transmission, and medications preventing the adverse effects of malaria in pregnant women and young children of age group 6–12 weeks old.^22^

Although the European Medicines Agency (EMA) gave the RTS, S/AS01 vaccine a good regulatory evaluation in July 2015, the World Health Organization (WHO) nonetheless suggested a large‐scale pilot research be conducted before recommending the vaccination for children aged 5–17 months old in October 2015. As of the present year, the vaccine has been the subject of large‐scale pilot research involving hundreds of thousands of young children of age group 6–12 weeks old in Ghana, Kenya, and Malawi. In all three countries, WHO partnering with the ministry of health and other research partners is now assessing the safety and impact of a large‐scale RTS, S/AS01 pilot program alongside a GSK‐led Phase 4 study in areas of moderate to high malaria transmission. This pilot study will examine the vaccine's effect on all‐cause mortality and its connection to particular adverse effects (febrile convulsions, meningitis, and cerebral malaria) to assess if the three‐dose vaccination series with booster can be provided via normal healthcare systems.^23^ Transmission‐blocking vaccines that target the sexual stage of the parasite's development in the mosquito and malaria mRNA vaccines are only two of the many malaria vaccine candidates now in research or clinical trials. The world's foremost authorities in public health have come together to create a Malaria Vaccine Technology Roadmap to speed up research and development of a viable malaria vaccine. The following targets for malaria vaccines by 2030 will be developed as part of the strategic objectives: Assuring the complete eradication of malaria through mass vaccination campaigns in multiple settings using malaria vaccines that reduce transmission and human malaria infection requires the provision of malaria vaccines with protective efficacy of at least 75% against clinical malaria in areas where malaria transmission is ongoing.^24^

### 3.2 Acceptance of malaria vaccine

The recent RTS, S malaria vaccine is a remarkable milestone towards the eradication of malaria, but the level of acceptance, especially in low and middle‐income countries, may pose an obstacle that must be overcome for effective implementation of the project. Historically, poor vaccination campaigns in different regions, including African and Asian territories, have prompted reservations regarding the successful adoption of malaria vaccines in endemic regions.^10^ For example, Nigeria formerly had the largest number of polio cases, which was blamed on the country's low vaccination coverage and compliance. Similarly, Pakistan and Afghanistan are also blamed for the failure to eradicate polio due to ineffective vaccination programs.^25^ The malaria vaccine has shown a very good response in several African countries, and the majority of parents are willing to administer the recommended dosage to their children.

Numerous studies conducted in different regions of Africa have evaluated the acceptance rates and participants’ attitudes toward the malaria vaccine. A cross‐sectional survey based in the community carried out by Asmare et al.^7^ revealed 32.3% of participants were in favor of immunizing their kids. According to research by Mtenga et al.^8^ 84.2% of people accepted the malaria vaccination. In this study women who were mothers and had a favorable impression of vaccinations made up most study participants. According to the mothers, vaccinations were crucial for disease prevention, disease severity reduction, and lower medical costs.^8^ In another study carried out in a remote Nigerian community, the malaria vaccination acceptance rate was found to be 91.6%.^9^ In the study by Ojakaa et al.,^10^ interviews from a standard questionnaire were done with 2003 caretakers at 695 randomly selected health facilities throughout Kenya. Around 88% of carers responded that they would support the vaccine for both a young child in their community and for their child, while approximately 7% of respondents did not know whether they would support the vaccine and 5% would not.^10^ Mothers, however, made up 90% of the carers in this study who visited the health facility with their children. In a similar study, 97% of participants with children under the age of 10, said they would want their child immunized against malaria if a safe and effective vaccine were available.^11^ However, according to the poll, 79% of participants said they had previously had a vaccination for at least one disease,^11^ which indicates that a successful prior vaccination history is essential for effective vaccination acceptance. Comparable research in Nigeria^13^ found that up to 88.2% of respondents had a favorable opinion of the vaccine, of which 65.2% had a very favorable perception.

This demonstrates that while malaria vaccine acceptability in African countries is good, there are still areas where vaccine acceptance is subpar.^26^ In contrast, the COVID‐19 vaccine acceptance rates were found to be 51%, 60%, 34%, and 63% in Ethiopia, Tanzania, Nigeria, and Kenya, respectively.^26^, ^27^, ^28^, ^29^ This disparity shows that the acceptance rate of the malaria vaccine is relatively higher than the COVID‐19 vaccination. This might be due to the fact that malaria has been a known disease for several decades and has caused millions of deaths, with children accounting for a sizable fraction of those deceased. On the other hand, COVID‐19 is a relatively new disease, and several myths are circulating the world due to emergency measures taken by governments all over the world and the economic halt.^17^

### 3.3 Personal beliefs and rumors

Another important hurdle to vaccination acceptance is the belief that vaccines contain harmful substances and transmit disease rather than protecting, as well as fear that the active elements in the injectable vaccine may harm their children. According to research, parents are less likely to vaccinate their children if they have to travel a great distance to get their shots, wait in a long line, or try to visit at off‐peak hours because of a lack of available appointments.^8^, ^19^ Besides that, a fragile healthcare system with inadequate facilities, understaffed hospitals, poor communication between healthcare workers and patients, and a lack of faith in the local healthcare system are all contributing factors to the low vaccination rate.^28^

In the study by Asmare et al.^7^ the main reason for participants not being willing to vaccinate their child is because participants thought if not given orally it may paralyze the child. In another study,^11^ participants described the same types of fears and rumors about general vaccines that they expressed as personal hesitancies, including adverse effects, the idea that vaccinations could be harmful or meant to cause sickness, and fear of needles or injections. In a qualitative study conducted in Ghana,^15^ rumors were found to influence attendance at vaccination campaigns. Some participants also had the perception that a malaria vaccine already exists.^15^ Another study in Kenya revealed participants had pre‐existing concerns and rumors about KEMRI being “devil worshipers.”^16^

### 3.4 Perception of malaria and malaria vaccine

Acceptance and adoption are just as crucial as effectiveness when introducing new treatment procedures to society.^11^ Individuals’ understandings of ailments and the desirable impacts of therapies that aim to prevent diseases, according to the health belief model, are major drivers of people's attitudes toward health interventions.^30^ Historically, the failure of an approved vaccine in a region is always due to a lack of community participation and perception. Recently, there has been an increase in the value placed on public involvement in the dissemination and application of cutting‐edge clinical research and community‐based preventative methods. Nevertheless, several obstacles make it difficult to make advantage of community participation, such as locating the relevant stakeholders and gauging their degree of engagement.^16^ A study in Nigeria^13^ revealed that awareness of malaria as a public health problem was high (89.8%), but awareness about a prospective malaria vaccine was not high (48.2%). In another study, there seemed to be less understanding of the purpose of the trial.^16^ The majority of the respondents (60.2%) strongly agreed that the malaria vaccine would prevent malaria.^13^ About 61.4% strongly agreed that everyone should receive the malaria vaccine.^13^

In general participants with higher knowledge about the vaccine have been found to have higher odds of accepting vaccination. There are still several factors causing hinder the maximum attainable coverage of the malaria vaccine that can prevent thousands of deaths. Literature has shown that the major factors contributing to hesitancy towards malarial vaccines include inadequate community engagement as the authorities fail to engage with the public. People do not have sufficient knowledge about vaccines, their efficacy, mechanism of action, and significance, which leads to the denial of immunization programs.^28^

### 3.5 Socioeconomic issues

Financial instability can be a significant barrier to healthcare access and is often linked to lower rates of vaccination. People who are struggling financially may have limited access to healthcare facilities, including vaccination centers, or may prioritize other expenses over‐vaccination. Additionally, those who are uninsured or underinsured may face higher out‐of‐pocket costs for vaccinations, making it more difficult for them to afford the vaccine.

In a study,^7^ participants asked for the vaccination to be provided without charge, indicating that they might not be able to afford the price or that they are unwilling to pay for the vaccine.^7^ Just 40.6% of respondents, according to another research, said they would be prepared to pay for the vaccination, of which 64% said they would do so to make it easily accessible. On the other hand, the plurality (45.1%) of those who may not be willing to pay for the vaccination said that the price would not likely be within the means of most households.^13^

### 3.6 Mode of administration, dosage, and side effects

The vaccine's viable administration route is crucial to its widespread use. The RTS, S/AS01 immunization vaccine is stored and administered similarly to other childhood vaccines. This facilitates the distribution of the vaccination even in nations with limited economic infrastructure. The World Health Organization (WHO) recommended testing a four‐dose regimen of RTS, S/AS01 in areas of Africa with moderate to high malaria transmission. The recommended protocol for administering the malaria vaccine involves administering three initial doses at intervals of at least 4 weeks, followed by a fourth dose 15 to 18 months after the third dose. The first dose should be given as close to 5 months of age as possible, and the third dose should be completed by the time the individual reaches 9 months of age. Additionally, it is permissible to administer RTS, S/AS01 in conjunction with other vaccines within the framework of a national immunization program.^28^ Previous studies have shown that RTS, S/AS01 may be safely administered with the pneumococcal conjugate immunization and the rotavirus vaccine. Coadministration of RTS, S/AS01 with vitamin A between 6 and 7.5 months of age, and with yellow fever and measles, rubella, and rabies vaccines around 9 months of age, did not affect the immunological response to immunizations.^31^

Vaccine effectiveness was shown to be significantly lower in clinical trials including infants aged 6–14 weeks compared to children aged 5–17 months.^22^ The low efficacy of the RTS, S/AS01 vaccination in babies was supposedly attributable to their underdeveloped immune systems and the interference of maternal antibodies.^22^ The RTS, S/AS01 vaccination may not be very effective, but it does have some positive effects on people's health all the same. To put it another way, for every 1000 children who had all four recommended immunizations, 1744 instances of malaria were averted. Phase III clinical tests conducted under the direction of the WHO found that four doses of the vaccine would prevent 484 deaths per 100,000 vaccinated children and 116,480 instances of malarial illness.^31^ Although not high in incidence, It is reported that RTS, S/A01 vaccine has various adverse effects including, but not limited to meningitis and cerebral malaria cases.^32^ Increased female mortality has also been observed in malaria vaccination groups.^33^ Increased incidence of pneumonia, anemia, febrile convulsions, and gastroenteritis has also been observed in all age groups including infants (Table 2).^37^

**Table 2:** Summary of the studies showing the mode of administration, dosage, and side effects of the malaria vaccine.

Article	Year	Journal	Outcomes
Final findings of Phase 3, individually randomized, controlled study examining the efficacy and safety of the RTS, S/AS01 malaria vaccine with and without a booster dose in babies and children in Africa.^22^	2015	The Lancet	In both age groups, the effectiveness was enhanced once a booster dose was given. Meningitis was identified as a serious adverse event (SAE) in 22 children. Within 7 days after the RTS, S/AS01 booster, 2.2 per 1000 doses of the vaccine were associated with generalized convulsive seizures in newborns, whereas the rate was 2.5 per 1000 doses in children. The vaccine might make a substantial contribution to malaria control, particularly in high‐transmission areas, when used in conjunction with other effective control measures.
CNS infection safety signal of RTS, S/AS01 and possible association with rabies vaccine.^34^	2016	The Lancet	Children aged 5–17 months who received the intervention experienced an increased incidence of meningitis than age‐matched controls.
RTS, S Malaria Vaccine and Increased Mortality in Girls.^33^	2016	American Society for Microbiology Journals	Increased risk of severe malaria in females is associated with RTS, S 1. (Malaria mortality ratio, 1.90 [0.82–4.37]) In children who had severe malaria, RTS, S was linked to a case fatality ratio that was two times greater.
The Future of the RTS, S/AS01 Malaria Vaccine: An Alternative Development Plan.^35^	2016	PLOS Medicine	In low‐endemicity areas, adding vaccination alongside aggressive malaria elimination programs could be the key to stopping transmission. The falciparum malaria vaccine candidate with the greatest global development progress is RTS, S/AS01. The antibody response to the intradermal rabies vaccine given for pre‐exposure immunization may be decreased by the concurrent use of chloroquine.
Immunogenicity and safety of the RTS, S/AS01 malaria vaccine co‐administered with measles, rubella and yellow fever vaccines in Ghanaian children: A Phase IIIb, multicenter, non‐inferiority, randomized, open, controlled trial.^36^	2020	Vaccine	RTS, S/AS01 can be administered along with vitamin A, the measles‐rubella, and yellow fever vaccines. When administered together, the vaccine's safety profiles were clinically acceptable. Children responded effectively to all vaccines, whether they were delivered alone or co‐administered with other vaccines.

## 4 DISCUSSION

The development of the RTS, S/A01 vaccine defines a major stride in the development of a vaccine for malaria. We highlighted the topic from three perspectives of acceptability, availability, and feasibility. From the perspective of acceptability, the health belief model states that people's perceptions of diseases and the benefits of interventions to prevent them play a major role in how they respond to health interventions.^38^ Previous studies in sub‐Saharan Africa have found that malaria is seen as a major public health problem.^39^ This suggests that vaccination against malaria is considered important by the population in this region. However, several challenges may affect vaccine acceptance, such as inadequate community engagement, concerns about side effects, and issues with the delivery and quality of healthcare services. Improving community engagement, providing information about the safety of the vaccine, and increasing the quality of healthcare services are crucial for boosting vaccine acceptance.^15^ Additionally, limited access to quality healthcare services and financial barriers may also affect vaccine acceptance. It is also important to note that while making vaccination free of charge and using incentives may boost attendance at vaccination sessions, the sustainability of these methods is questionable.^19^ Furthermore, factors that negatively affect access to vaccination, such as lack of transportation or long distances to healthcare facilities, also need to be addressed. Additionally, addressing the perception of completion of the vaccination calendar, as reported in a study conducted in Ghana, where hospital attendance of women and children drops after the age of 9 months, is crucial for maintaining vaccine acceptance.^15^ Another important aspect to consider when addressing vaccine acceptance in sub‐Saharan Africa is cultural and religious beliefs. Studies have shown that some communities in the region may have specific cultural or religious beliefs that may affect their willingness to accept vaccination.^12^ For example, some communities may view vaccination as a violation of their traditional beliefs or as a form of Western intervention.^8^ It is, therefore, crucial for healthcare providers and researchers to engage with these communities, understand their specific cultural and religious beliefs, and address any concerns they may have. This could be done by involving local leaders, religious leaders, and traditional healers in the implementation of vaccination programs. Additionally, addressing vaccine hesitancy among healthcare providers is also crucial for increasing vaccine acceptance in the region. Some healthcare providers may have misconceptions or concerns about vaccines that can affect their ability to effectively communicate the benefits of vaccination to their patients. Addressing these concerns through education and training programs can help increase the confidence of healthcare providers in the safety and efficacy of vaccines and improve their ability to persuade patients to accept vaccination. While the overall acceptance of malaria vaccines in sub‐Saharan Africa is high, there are still various challenges and barriers that need to be addressed to ensure the success of vaccination programs in the region. A multifaceted approach that includes effective community engagement, addressing concerns about side effects, improving the delivery and quality of healthcare services, as well as cultural and religious considerations, and addressing vaccine hesitancy among healthcare providers is needed to ensure that the benefits of vaccination reach everyone in need.^40^

Traditional cultural practices and beliefs, single family members making healthcare decisions in households, and the relationship of the caregiver with the child are considered minor factors towards vaccine hesitancy.^17^, ^19^, ^29^ Vaccination reluctance can be reduced through a variety of strategies, the most prominent of which is the use of adaptable and extensive communication models and trusted sources to deliver health information to communities; the promotion of community participation at both the national and district levels; and the introduction of new vaccine services alongside existing health services. Careful consideration of the sociocultural context of each community is essential for the effective and efficient implementation of the malaria vaccine program.^40^ Vaccine acceptance research has repeatedly pushed for programs to improve the public's knowledge and opinion of vaccines' effectiveness. The success of immunization programs depends not only on their clinical effectiveness but also on how the public views them. Higher rejection rates may be attributed to a lack of community involvement, misunderstandings, and knowledge. Vaccination reluctance can be reduced through a variety of strategies, the most prominent of which is the use of adaptable and extensive communication models and trusted sources to deliver health information to communities; the promotion of community participation at both the national and district levels; and the introduction of new vaccine services alongside existing health services. Careful consideration of the sociocultural context of each community is essential for the effective and efficient implementation of the malaria vaccine program.^40^

From an availability standpoint, the vaccine may not be as quickly available to meet the demands. The World Health Organization's (WHO) endorsement of the RTS, S malaria vaccine is a pivotal development in the quest to provide immunity to the malady of malaria for children in impoverished regions. However, the production and availability of the vaccine remain constrained, resulting in a scenario wherein the majority of affected children will not have access to it in the immediate future. It is imperative to acknowledge that the demand for the vaccine is substantial, with projections indicating that a minimum of 100 million to 160 million doses per annum are necessary to cater to the malaria‐endemic areas in sub‐Saharan Africa alone. The process of transferring the technology and augmenting production to meet this demand will be a gradual one, as evidenced by the drug manufacturer's commitment to producing a maximum of 15 million doses per annum until 2028, following pilot programs conducted in 2019. However, it is unlikely that the company will be able to manufacture more than a few million doses annually before 2026, owing to the lack of funding and commercial viability.^21^, ^22^ The unfortunate reality is that malaria, a disease that disproportionately affects low‐ and middle‐income countries (LMICs) that lack robust health infrastructure, has resulted in a lack of incentive for vaccine manufacturers to invest in the development of malaria vaccines. This is due in large part to the fact that these manufacturers tend to focus their efforts on markets within the industrialized world, where the profit potential is often greater. Additionally, the stringent regulations imposed by national vaccine licensing authorities further exacerbate the issue by significantly increasing the cost of the clinical development pathways required for vaccine licensure. This, in turn, makes it more financially challenging for manufacturers to recoup their investments in the development of new vaccines unless they receive support from nongovernment organizations or are made available at subsidized rates through public–private partnerships. In summary, the combination of a lack of incentive for vaccine manufacturers to invest in malaria vaccines, and the high costs associated with clinical development pathways and regulatory compliance, makes it difficult to develop and bring new malaria vaccines to market.^41^

From the spectacle of feasibility, the malaria vaccine, RTS, S/AS01, developed by GlaxoSmithKline Biologicals, has been found to be feasible. It has been shown to lower mortality in clinical settings and has been recommended for use by the World Health Organization (WHO) in regions with moderate to high malaria transmission.^42^ The vaccine's administration is similar to other childhood vaccines, making it easy to distribute even in areas with limited economic infrastructure. It has also been found to be cost‐effective and can be safely administered with other vaccines.^28^ However, the vaccine's effectiveness is lower in infants aged 6–14 weeks due to their underdeveloped immune systems and interference of maternal antibodies.^22^ Overall, it has been found to prevent 484 deaths per 100,000 vaccinated children and 116,480 instances of malarial illness in Phase III clinical tests.^31^ In addition, the RTS, S/AS01 vaccine has been shown to have a long‐term impact on reducing malaria cases. The WHO recommends a four‐dose regimen of the vaccine, with the first dose given as close to five months of age as possible and the third dose completed by nine months of age. The fourth dose is given 15–18 months after the third dose. The vaccine can also be administered in conjunction with other vaccines as part of a national immunization program.^28^ Overall, the RTS, S/AS01 malaria vaccine is a promising development in the fight against malaria. While it may not be as effective as some other vaccines, it has been shown to have a significant impact on reducing malaria cases and deaths, especially in areas with moderate to high transmission.^31^ The vaccine's ease of administration and low cost make it a viable option for use in low and middle‐income countries, where malaria is a major public health concern.^43^, ^44^

## 5 CONCLUSION

In conclusion, the development of the RTS, S/A01 vaccine for malaria is a significant stride in the fight against the disease. However, there are several challenges that need to be addressed to ensure the success of vaccination programs. From the acceptability perspective, issues such as inadequate community engagement, concerns about side effects, and issues with the delivery and quality of healthcare services can affect the acceptance of the vaccine. Additionally, limited access to quality healthcare services and financial barriers may also affect vaccine acceptance. From the feasibility standpoint, factors such as lack of transportation or long distances to healthcare facilities and the perception of completion of the vaccination calendar can affect the feasibility of the vaccine. To overcome these challenges, addressing the accessibility and proximity of healthcare facilities and addressing the perception of completion of the vaccination calendar can improve the feasibility of the vaccine. Lastly, the availability of the vaccine is also a major concern as it may not be readily available to meet the demands. To overcome this, a multifaceted approach that includes effective community engagement, addressing concerns about side effects, improving the delivery and quality of healthcare services, as well as cultural and religious considerations, and addressing vaccine hesitancy among healthcare providers is needed to ensure that the benefits of vaccination reach everyone in need.

## AUTHOR CONTRIBUTIONS

**Hassan Mumtaz**: Writing—review & editing. **Abdullah Nadeem**: Conceptualization; writing—original draft. **Wajeeha Bilal**: Writing—review & editing. **Farrukh Ansar**: Formal analysis. **Saira Saleem**: Writing—review & editing. **Qaisar Ali Khan**: Writing—review & editing. **Tamara Tango**: Data curation. **Christopher Farkouh**: Formal analysis; writing—original draft. **Naod F. Belay**: Formal analysis; Writing—original draft. **Ravina Verma**: Project administration; writing—original draft. **Matthew Farkouh**: Writing—review & editing. **Muhammad Saqib**: Writing—review & editing.

## CONFLICT OF INTEREST STATEMENT

The authors declare no conflict of interest.

## Data Availability

Data is available upon reasonable request made to the first author Hassan Mumtaz (hassanmumtaz.dr@gmail.com).
